# Bio-functionalization and *in-vitro* evaluation of titanium surface with recombinant fibronectin and elastin fragment in human mesenchymal stem cell

**DOI:** 10.1371/journal.pone.0260760

**Published:** 2021-12-16

**Authors:** Bo-Hyun Park, Eui-Seung Jeong, Sujin Lee, Jun-Hyeog Jang

**Affiliations:** Department of Biochemistry, Inha University School of Medicine, Incheon, Korea; Università degli Studi della Campania, ITALY

## Abstract

Titanium is a biomaterial that meets a number of important requirements, including excellent mechanical and chemical properties, but has low bioactivity. To improve cellular response onto titanium surfaces and hence its osseointegration, the titanium surface was bio-functionalized to mimic an extracellular matrix (ECM)-like microenvironment that positively influences the behavior of stem cells. In this respect, fibronectin and elastin are important components of the ECM that regulate stem cell differentiation by supporting the biological microenvironment. However, each native ECM is unsuitable due to its high production cost and immunogenicity. To overcome these problems, a recombinant chimeric fibronectin type III_9-10_ and elastin-like peptide fragments (FN9-10_ELP_) was developed herein and applied to the bio-functionalized of the titanium surface. An evaluation of the biological activity and cellular responses with respect to bone regeneration indicated a 4-week sustainability on the FN9-10_ELP_ functionalized titanium surface without an initial burst effect. In particular, the adhesion and proliferation of human mesenchymal stem cells (hMSCs) was significantly increased on the FN9-10_ELP_ coated titanium compared to that observed on the non-coated titanium. The FN9-10_ELP_ coated titanium induced osteogenic differentiation such as the alkaline phosphatase (ALP) activity and mineralization activity. In addition, expressions of osteogenesis-related genes such as a collagen type I (Col I), Runt-related transcription factor 2 (RUNX2), osteopontin (OPN), osteocalcin (OCN), bone sialo protein (BSP), and PDZ-binding motif (TAZ) were further increased. Thus, *in vitro* the FN9-10_ELP_ functionalization titanium not only sustained bioactivity but also induced osteogenic differentiation of hMSCs to improve bone regeneration.

## Introduction

Implantable materials play an important role in dental and orthopedic procedures [1]. In particular, titanium is predominantly used in the biomedical field due to its excellent biocompatibility and mechanical properties; however, it does not promote bioactivity towards bone regeneration by itself [1–3]. Therefore, a key strategy for osteogenic stimuli is to enhance cellular responses by biofunctionalization of titanium surfaces with biomolecules [4].

In this respect, the extracellular matrix (ECM) is a biomolecule that provides a microenvironment to induce specific signaling pathways in stem cells, resulting in positive effects [5,6]. However, native ECM proteins share several common drawbacks such as immunological rejection by the host and high production costs [7]. Thus, an alternative approach is to utilize the recombination of biomimetic ECMs and biofunctionalization of titanium surfaces with recombinant ECM proteins [8–10].

Fibronectin (FN) is an important component of the ECM and is crucial for the regulation of cellular functions such as cell migration, proliferation, differentiation, and viability [11–13]. As shown by the results, the interaction between FN and integrin mediates cell binding and is important during osteoblast differentiation for bone formation [14–16]. Structurally, FN is a protein dimer in which two nearly identical monomers are linked by a pair of C-terminal disulfide bonds, and each FN subunit consists of homologous repeats of three types of modules (FNⅠ, FNⅡ, FNⅢ) [17]. The Arg-Gly-Asp (RGD) sequence located in module Ⅲ tenth type is the main cell adhesion site and binds α_5_β_1_ integrin, which is the most common FN-receptor [18]. Meanwhile, the Pro-His-Ser-Arg-Asn (PHSRN) sequence located in module III ninth type is a synergy site, which promotes the FN-α_5_β_1_ integrin interaction [19]. The FNⅢ 9−10 sequence is widely used in biomedical applications as a growth factor that improves cellular activities such as cell adhesion, proliferation, and osteoblast differentiation [20–22].

Elastin is an ECM protein found in connective tissues and blood vessels and is important for the mechanical properties of extensible ligaments and organs (like lungs and aorta) [23]. Elastin is composed of a matrix of cross-linked tropoelastin, which accounts for 80% of hydrophobic domains e.g., the common repeating sequence of valine, proline, glycine, alanine (VGVAPG) [23–25]. However, insoluble elastin is difficult to use as a biomaterial because its structure contains a wide range of cross-linked domains [26,27]. By contrast, the elastin-like-polypeptides (ELPs) are a class of bioengineered peptide polymers, derived from hydrophobic residues of human tropoelastin [23,28]. The ELPs consist of multiple repeating pentapeptide sequences of Val-Pro-Gly-Xaa-Gly, where Xaa indicates a guest residue that can be any amino acid except proline and is either soluble or insoluble depending on the reverse transition temperature [28–31]. The ELPs not only exhibit biocompatibility including favorable mechanical and viscoelastic properties, but also improve the adhesion, proliferation, and differentiation of cells [32,33].

Human mesenchymal stem cells (hMSCs) have the potential to differentiate into various lineages such as bone, fat, chondrocyte, muscle, neuron, and liver cells under specific *in vitro* conditions and can be isolated from a variety of tissues, including umbilical cord, endometrial polyps, menses blood, bone marrow, and adipose tissue [34]. In particular, hMSCs can be differentiated into osteoblast-like cells by regulating the microenvironment with osteogenic stimuli such as bone morphogenic protein (BMP)-2 [35]. Recently, many studies have reported that modified titanium promotes osteogenic differentiation of various stem cell types [36,37]. Naddeo P et al. studied the earlier osteogenic differentiation and proliferation of hMSCs through osseoinduction on a titanium surface for osseointegration between bone and implant [38]. Conserva E et al. reported that titanium surface modification affects the expression of FasL in MSCs, exhibiting various immunomodulatory properties [39]. Thus, hMSCs are suitable candidates for new bone formation and bone transplantation for human tissue regeneration in tissue engineering.

The purpose of present study was to investigate the effect of FN9-10_ELP_ on the adhesion, proliferation, and differentiation of hMSCs on titanium for bone tissue engineering. An additional purpose was to examine FN9-10_ELP_’s the release kinetics to assess the sustainability of FN9-10_ELP_ from titanium. The results of the present study demonstrated that FN9-10_ELP_ can promote the osteogenic differentiation of hMSCs on titanium disc, and the release profiles indicate sustained biological activity of hMSCs on the titanium during the period of osteogenesis.

## Materials and methods

### Construction and extraction of recombinant FN9-10_ELP_

The FN9-10_ELP_ fusion protein was produced as previously described by Lee et al [40]. Briefly, the ELP sequence was synthesized (Genotech, Daejeon, Korea) and cloned into the pBAD-His-FN9-10 expression vector using the restriction enzymes *Sac*Ⅰ and *Hind*Ⅲ. The FN9-10_ELP_ construct was transformed into *E*. *coli* TOP10, as the expression host.

To express FN9-10_ELP_, the transformed TOP10 cells were incubated overnight at 37°C in lysogeny broth (LB) medium containing 100 μg·mL^−1^ ampicillin (LB-Amp^+^). When the culture medium reached OD_600_ = 0.6, induction was performed for 6 h by adding 0.1% (w/v) L-arabinose to induce protein expression. The bacteria were harvested and pelleted by centrifugation at 6000 *g* for 10 min, lysed in NaCl-Tris-EDTA (STE) buffer and sonicated. The soluble extract was centrifuged at 13,000 *g* for 2 x 10 min. The collected supernatant was purified by passing through a chromatography column containing a nickel-nitrilotriacetic acid resin (Invitrogen, Carlsbad, CA).

The recombinant FN9-10_ELP_ purity was evaluated using coomassie blue staining on 12% (v/v) SDS-PAGE gels. In addition, the molecular weight and expression of recombinant FN9-10_ELP_ were confirmed by western blotting using the horseradish peroxidase (HRP) conjugate of monoclonal anti-polyhistidine antibody (His antibody, sc-8036 HRP, Santa Cruz Biotechnology, Santa Cruz, CA, USA).

### Cell collection and ethical issues

The hMSCs were derived from nasal inferior turbinate tissue obtained from preoperative patients with the approval of the Institutional Review Board of Seoul St. Mary’s Hospital, the Catholic University of Korea (approval number KC08TISS0341) and used in this study. Written consents of donors were obtained, documented, and witnessed. The isolation of hMSCs were reported in detail in previous studies [41].

### Cell culture and preparation of the titanium discs

The hMSCs were cultured in α-minimal essential medium (α-MEM; Welgene, Gyeongsan, Korea) containing 10% (v/v) fetal bovine serum (FBS; Welgene, Gyeongsan, Korea), 100 μg·mL^−1^ streptomycin, and 100 U/mL penicillin G sodium at 37°C under 5% CO_2_ atmosphere. The cultured cells were detached using 0.25% trypsin-EDTA and sub-cultured in fresh cell dishes (diameter 100 mm).

The titanium discs (25mm diameter and 1mm thickness) are consisted of grade 2 commercial titanium sheet. Before coating with the fusion protein, all the titanium discs were degreased and passivated by washing with acetone, 2% ammonium fluoride, and 2% hydrofluoric acid-10% nitric acid. Finally, the discs were autoclaved and dried in an oven at 50°C and stored at room temperature (RT).

### Protein attachment assay

The protein attachment to the titanium surface was confirmed by an ELISA using a His antibody probe. To confirm the maximum concentration of FN9-10_ELP_ that binds to the titanium discs, the discs were immersed overnight in various concentrations (0−25 μg) of FN9-10_ELP_ made up to a volume of 30 μL/disc in 6-well plates at 4°C. The nonspecific binding capacity was reduced by blocking with 2% (w/v) bovine serum albumin (BSA, Bovogen, Australia) solution. After washing with Dulbecco’s phosphate-buffered saline (DPBS; Welgene), 400 μL of monoclonal anti-polyhistidine antibody was diluted 1:1000, added to the discs, and reacted at RT for 1h. After washing with tris-buffered saline/polysorbate 20 (TBST; LPS Solution, Daejeon, Korea), 200 μL of 3,3′,5,5′-tetramethylbenzidine (TMB) substrate solution (1-Step Ultra TMB ELISA, Thermo, USA) was added to the discs and reacted at RT for 30 min in the absence of light. Next, 100 μL of the reaction solution and stop solution (2M sulfuric acid) were added to a 96-well plate. The absorbance of each sample was quantified at 450 nm using a microplate reader (Biotech, Seoul, Korea).

### *In vitro* release profile of FN9-10_ELP_ from titanium discs using an ELISA

The release profile from the FN9-10_ELP_-coated titanium discs was measured for 28 days by ELISA-based retention assay using a His antibody probe. The titanium discs were immersed overnight in 10 μg·mL^−1^ FN9-10_ELP_ made up to a volume of 500 μL/disc in a 6-well plate at 4°C. The discs were then incubated in PBS (Welgene, Gyeongsan, Korea) at 4°C and replenished with fresh buffer every day. The collected discs were then dried using compressed air and blocked with 2% (w/v) BSA solution for 90 min. After washing with DPBS, horseradish peroxidase (HRP) conjugate of a His antibody (dilution at 1:1000) was reacted at RT for 1 h. The unbound antibody was removed with TBST, then 200 μL of TMB substrate solution was added to the discs and incubated at RT for 30 min under dark conditions. To complete the reaction, 100 μL of the reaction solution and stop solution (2M H_2_SO_4_) were mixed. The degree of release of FN9-10_ELP_ attached to titanium discs was quantified using a microplate reader at 450 nm, with 5% (w/v) BSA solution as the control. The control profile was measured for 28 days using a Bradford protein assay. For this experiment the BSA coating and *in vitro* release using PBS were performed using the same procedure as above, then 50 μL of PBST (PBS + 0.1% Tween 20) was added to release the BSA from the collected discs, and reacted at RT for 30 min. Next, 50 μL of Coomassie Plus Assay Reagent was added and reacted for 30 min under dark conditions at RT. The degree of release of the control marker from the titanium discs was quantified using a microplate reader at 570 nm.

### Cell adhesion assay

The cell adhesion activity on the titanium discs was evaluated via a crystal violet assay. The titanium discs were immersed overnight in 10 μg·mL^−1^ FN9-10_ELP_ made up to a volume of 500 μL/disc at 4°C. The un-coated titanium discs were used as the control. All discs were blocked with 2% (w/v) BSA solution for 2 h. After washing with DPBS, hMSCs were seeded at a density of 1 × 10^5^ cells/disc and incubated at 37°C. To determine the optimal cell adhesion time, the cells were incubated for up to 150 min at 30 min intervals. After incubation, the discs were washed with DPBS, and the cells attached to the discs were fixed with 3.7% (w/v) formalin solution at RT for 30 min. The cells fixed on the discs were stained with 0.25% (w/v) crystal violet in 2% ethanol/water (Sigma, St Louis, USA) and washed with DPBS after 1 h. The stained cells were lysed by adding 2% SDS solution to the discs and transferred to a 96-well plate. The absorbance of each sample was measured at 570 nm using a microplate reader.

### Cell proliferation assay

The cell proliferation activity on the titanium discs was determined using an MTT assay, which measures cellular metabolic activity as an indicator of cell viability and growth. This assay was performed according to the manufacturer’s instructions (AMRESCO Inc, Solon, USA). First, the titanium discs were immersed overnight in 10 μg·mL^−1^ at 4°C, then hMSCs were seeded at a density of 1 × 10^4^ cells/disc and incubated for 4 and 8 days at 37°C. In addition, non-coated titanium discs were seeded with hMSCs as a control experiment. Each disc was washed with DPBS, then viable cells on the discs were incubated with 500 μL MTT solution (5 mg·mL^-1^ in DPBS) at 37°C in the dark. After 3 h of reaction, the MTT solution was removed, and the cells were lysed with 150 μL DMSO to dissolve the purple formazan crystals contained in the cells. The absorbance of each sample was determined at 570 nm using a microplate reader.

### Alkaline phosphatase (ALP) assay

Osteoblast differentiation on the titanium discs was investigated using an ALP assay, which measures the activity of ALP as an osteogenic marker expressed by early osteoblast during bone formation. The titanium discs were placed overnight in the presence or absence of 10 μg·mL^−1^ FN9-10_ELP_ at 4°C. Then, hMSCs were plated at a density of 5 × 10^3^ cells/disc and incubated for 5 and 10 days at 37°C. Next, the discs were rinsed with DPBS and the cells on the discs were lysed with 100 μL 0.1% Triton x-100 at RT for 30 min. After removing the insoluble materials by centrifugation at 4°C for 10 min, the ALP activity of the soluble cell lysate was evaluated using an alkaline phosphatase assay kit (Sigma). The absorbance of the colored reaction mixture was read at 405 nm using a microplate reader, and the ALP activity was normalized to the control (i.e., the non-coated titanium discs).

### Alizarin Red S staining

Late osteoblast differentiation on the titanium discs was determined by Alizarin Red S staining. Mineralization is known to late phase of osteogenic differentiation expressed by mature osteoblast during bone formation. The titanium discs were immersed overnight in 10 μg·mL^−1^ FN9-10_ELP_ at 4°C. Then, hMSCs were cultured at a density of 5 × 10^3^ cells/disc for 5 and 10 days. The discs were then rinsed with DPBS, fixed in 4% paraformaldehyde at RT for 30 min. Fixed hMSCs on the discs were stained using an Alizarin Red S solution (Sigma) overnight. Stained cells on the discs were washed with distilled water and destained with 250 μL of 10% cetylpyridinium chloride solution at RT for 30 min. The concentration of Alizarin Red S staining was quantified by measuring the absorbance at 450 nm using a microplate reader.

### RNA extraction and cDNA synthesis

For this experiment, hMSCs were incubated for 20 days on titanium discs coated with 10 μg·mL^−1^ FN9-10_ELP_. The total RNA was extracted using an Easy-spin RNA extraction kit (iNtRON, Seoul, Korea). The RNA purity was quantified by confirming the absorbance at 260nm and 280nm (A_260_/A_280_ ratio values of 1.9–2.1) with a NanoDrop 2000 spectrometer (Thermo Scientific). 2 micrograms of total RNA were reverse transcribed using a 20 μL reaction mixture including 50 units of SuperScript II reverse transcriptase (Invitrogen, Carlsbad, CA, USA), 0.5 μM of random hexanucleotide primers (Invitrogen), 40 units of RNaseOUT recombinant ribonuclease inhibitor (Invitrogen), 500 μM of dNTP mixture (Invitrogen), and 5 μM DTT. The reverse transcription was performed at 50°C for 1 h. Then, the reaction mixture was heated at 70°C for 15 min to stop the reaction. The cDNA product was stored at −20°C.

### Quantitative real-time PCR analysis

The mRNA expression of osteogenesis-related genes (Col I, RUNX2, OPN, OCN, BSP, and TAZ) was examined by quantitative real-time PCR. The primer sequences of these genes used in this experiment are listed in Table 1. All real-time PCR experiments were analyzed on an ABI Step One real-time PCR system (Applied Biosystem, Foster City, USA). The PCR was performed using a 20 μL reaction mixture including 0.1 μM of each primer, template cDNA, and 2x SYBR Green PCR master mix (Applied Biosystem, containing AmpliTaq Gold DNA polymerase in buffer, a dNTP mix, SYBR Green I dye, and 10 mM MgCl_2_). The PCR cycles were as follows: 1 cycle at 94°C for 10 min (enzyme activation), 40 cycles at 94°C for 15 s (denaturation), 60°C for 1 min (annealing), and 60°C for 1 min (extension).

**Table 1:** Sequence of primer used in the real-time PCR.

Genes	Forward primer	Reverse primer
**β-actin**	**5’-** **TGGCACCCAGCACAATGAAGAT** **-3’**	**5’-** **TACTCCTGCTTGCTGATCCA** **-3’**
**Col I**	**5’-** **GAAAATGGAGCTCCTGGTCA** **-3’**	**5’-** **ACCCTTAGCACCAACAGCAC** **-3’**
**RUNX2**	**5’-** **TTACCCCTCCTACCTGAGCC** **-3’**	**5’-** **GGTGTGGTAGTGAGTGGTGG** **-3’**
**OPN**	**5’-** **TGGATGACCAGAGTGCTGAA****-3’**	**5’-** **TTGGGGTCTACAACCAGCAT****-3’**
**OCN**	**5’-** **GACTGTGAGTTGGCTGA** **-3’**	**5’-** **AGCAGAGCGACACCCTAGAC** **-3’**
**BSP**	**5’-** **AGTACCAACAGCACAGAGGC** **-3’**	**5’-** **ACGCCCGTGTATTCGTACTC** **-3’**
**TAZ**	**5’-** **ACCGTGTCCAATCACCAGTC****-3’**	**5’-** **TGTAGCTCCTTGGTGAAGCA****-3’**

### Western blot analysis

The protein expression of osteogenic differentiation was investigated by western blotting. The hMSCs were cultured on titanium discs coated with 0 or 10 μg·mL^−1^ FN9-10_ELP_ for 20 days and then were lysed using a sodium dodecyl sulfate buffer. Extracted proteins were denatured by heating, then separated on 7.5 and 12% (v/v) SDS-PAGE gels and transferred to PVDF membrane. After blocking with 10% skim milk, the membrane was probed with mouse monoclonal Col I, OPN, and OCN antibody (dilution at 1:1000; Santa Cruz Biotechnology, Santa Cruz, CA, USA) at RT for 2 h. β-actin was used as the internal control. The blots were detected and quantified using a ChemiDoc Touch Imaging System (BIO-RAD, CA, USA).

### Statistical analysis

All experiments were performed in triplicate. The experimental data were reported as the mean ± standard deviation (SD). The statistical analysis was performed using the Student’s *t*-test. In all statistical analyses, a *p* value < 0.05 is recognized to be statistically significant for the difference between the two groups. (* p < 0.05, ** p < 0.01 and *** p < 0.001).

## Results

### FN9-10_ELP_ expression in *E*. *coli* and purification using a nickel-nitrilotriacetic acid resin

To construct the fusion FN9-10_ELP_, the sequence encoding FN9-10_ELP_ was cloned into a pBAD/His expression vector. This vector contains an ara BAD promoter (pBAD) that controls host expression and N-terminal polyhistidine for affinity purification and protein quantification.

**Fig 1:**
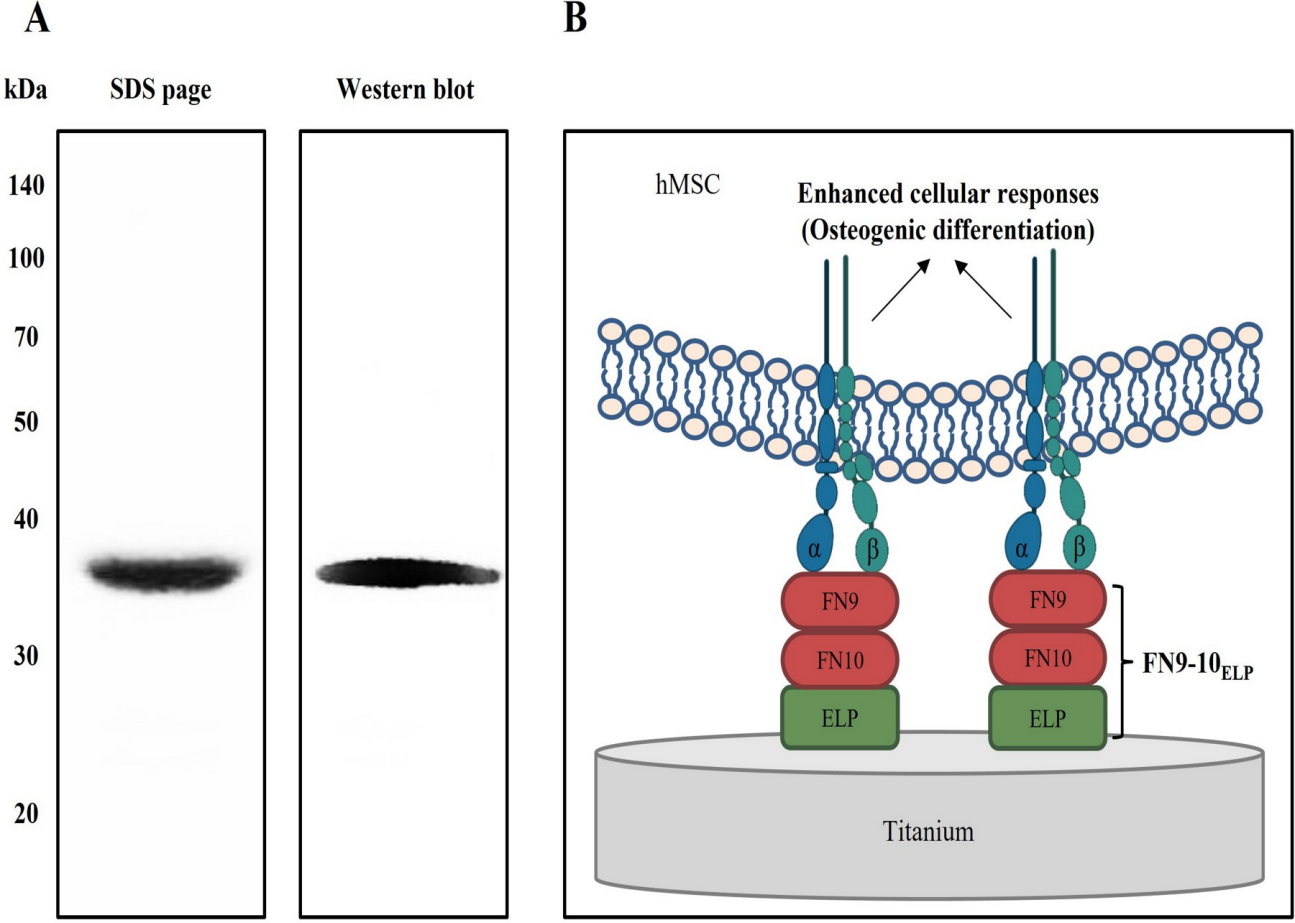
Expression of chimeric FN9-10_ELP_ and schematic illustration of bio-functionalization for enhanced cellular responses on titanium discs. (A) The purity and molecular weight of chimeric FN9-10_ELP_ were measured by 12% SDS-PAGE and Western blotting. The molecular weight shown is approximately 38 kDa. (B) Bio-functionalization of titanium discs using chimeric FN9-10_ELP_ to induce osteogenic differentiation of hMSCs.

The recombinant FN9-10_ELP_ was purified by affinity chromatography using an Ni-NTA column, and its purity was confirmed to be greater than 90% via Coomassie blue staining (Fig 1). In addition, the molecular weight of the recombinant FN9-10_ELP_ was determined to be approximately 38 kDa by western blotting.

### Attachment activity of FN9-10_ELP_ on titanium discs

**Fig 2:**
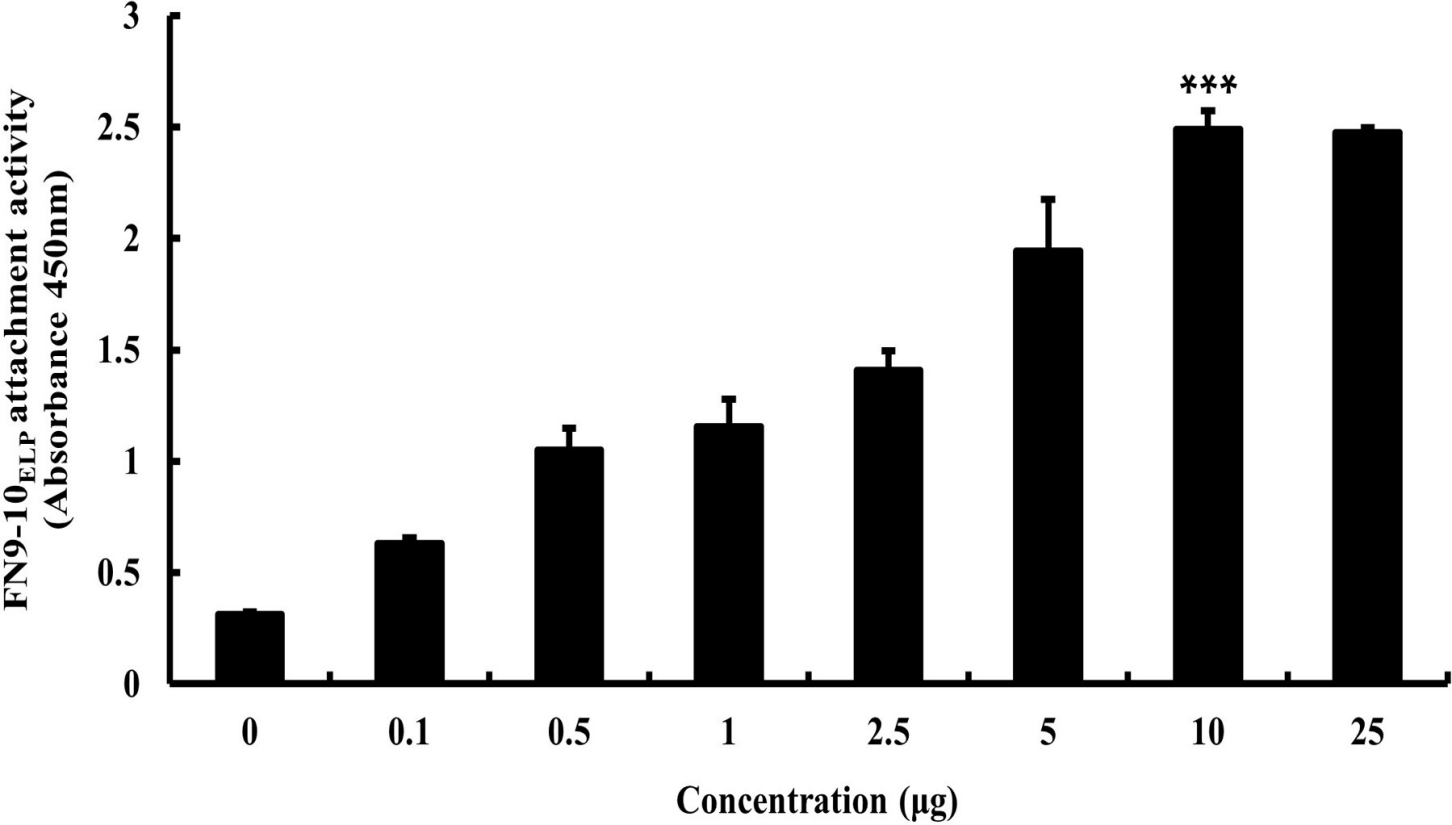
Protein attachment activity of FN9-10_ELP_ on the titanium discs. The titanium discs were immersed overnight in various concentrations of FN9-10_ELP_ (0−25 μg) in 6-well plates at 4°C. The absorbance of FN9-10_ELP_ was quantified by ELISA using a His-tag probe and reported the mean ± SD (n = 3). p< 0.001.

To confirm the adhesion of FN9-10_ELP_, titanium discs were coated with various concentrations (0−25 μg) of FN9-10_ELP_ overnight at 4°C and measured by the enzyme-linked immunosorbent assay (ELISA) using a His antibody probe as specified in the Materials and Methods Section. As shown in Fig 2, the attachment intensity of the FN9-10_ELP_ coated titanium disc increased with increasing proportion of FN9-10_ELP_ up to a maximum of 10 μg and remained constant thereafter (p < 0.001). These results indicated that the maximum concentration of FN9-10_ELP_ attached to the titanium discs was 10 μg. Thus, 10 μg of FN9-10_ELP_ was used in all further experiments to functionalize the surface of the titanium disc.

### Release profile of FN9-10_ELP_ from titanium discs

**Fig 3:**
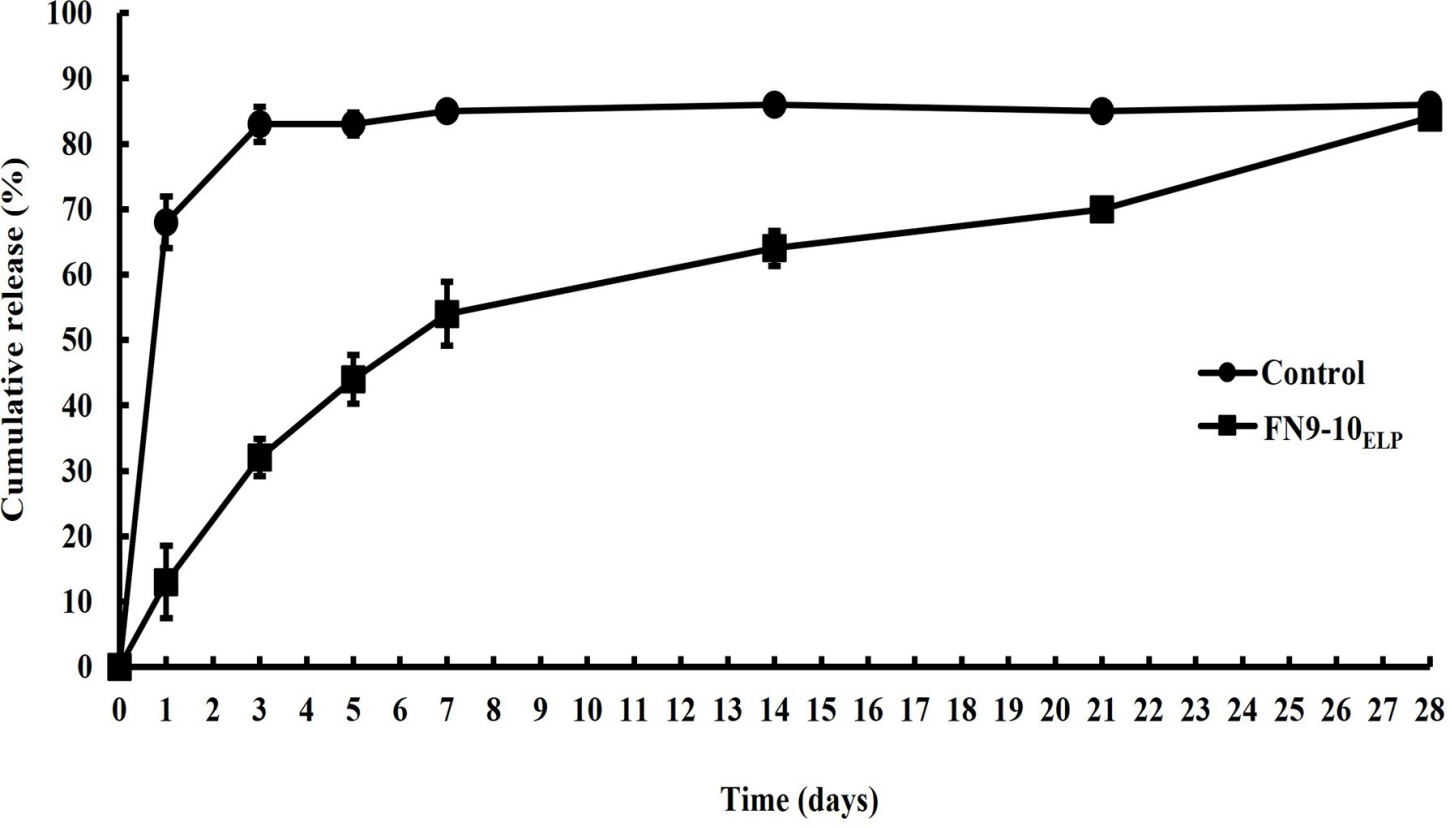
*In vitro* cumulative release profiles of FN9-10_ELP_ from titanium discs. The titanium discs were coated with FN9-10_ELP_ (10 μg·mL^−1^) and a control substance (5% BSA) in a volume of 500 μL/disc overnight at 4°C. The amount of FN9-10_ELP_ absorbed from titanium discs was measured using an ELISA. The cumulative release was evaluated as the initial attachment amount (100%). The Release profiles indicate the mean ± SD (n = 3).

To assess the release profile of the functionalized titanium discs, an ELISA-based retention assay was performed for 28 days using a His antibody probe. The FN9-10_ELP_ (10 μg·mL^−1^) and a control substance (5% BSA) were coated onto separate titanium discs overnight at 4°C, and the amount of released from the FN9-10_ELP_-coated titanium discs, and from the BSA-coated control discs, was quantified after immersion in phosphate-buffered saline (PBS) for up to 28 days (4 weeks). The amount of samples released from the titanium disc then was then calculated as the difference between the initial amount (100%) and the remaining amount. The results presented in Fig 3, indicated that the degree of attachment of FN9-10_ELP_ was initially high and gradually decreased over time without early burst effect. Thus, 54% of the FN9-10_ELP_ was released from the titanium discs during the first week of immersion, with a release rate of 7.7% per day. For subsequent 3 weeks, a release rate of about 1.4% per day was observed. By contrast, the release profile of BSA-coated (control) titanium discs exhibited an early burst pattern for up to 3 days, with about 83% of the BSA being released at a rate of 7.7% per day during this time. Moreover, the BSA was completely removed from the titanium discs after only one week. These results showed that the release profile of FN9-10_ELP_-coated titanium discs follows a sustained pattern over a period of 4 weeks and that biological activity is retained during this time.

### hMSCs adhesion effect of FN9-10_ELP_ on titanium discs

**Fig 4:**
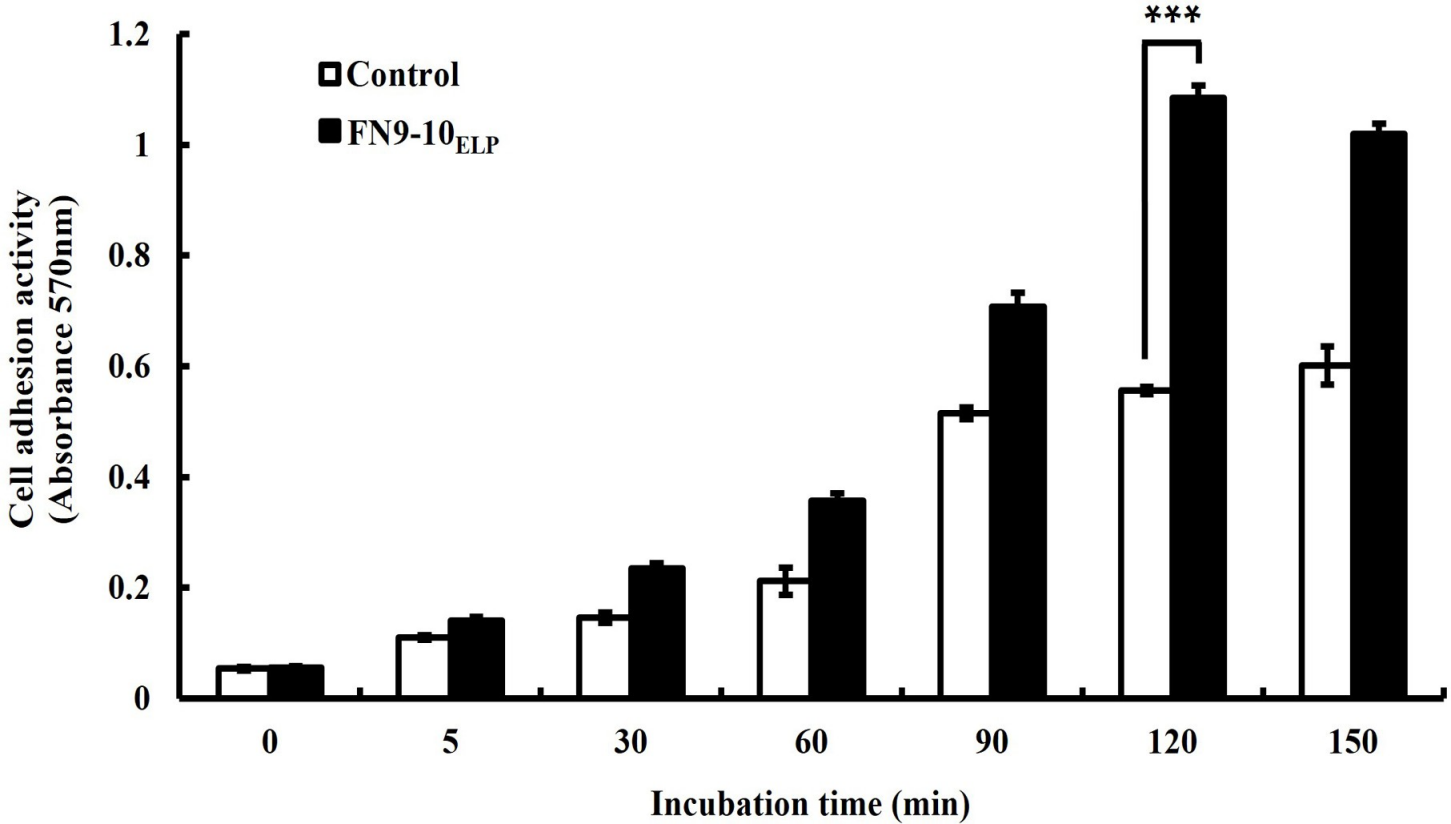
Cell adhesion activity of hMSCs according to incubation time on the FN9-10_ELP_-titanium discs. The titanium discs were immersed overnight in 10 μg·mL^−1^ of FN9-10_ELP_ at 4°C, and while the non-coated titanium discs served as the control. The hMSCs were seeded at a density of 1 × 10^5^ cells/disc on the titanium discs and incubated at 37°C for up to 150 min. The hMSCs adhesion activities were evaluated by crystal violet assay and are presented as the mean ± SD (n = 3). p < 0.001.

To determine the hMSCs adhesion effect of FN9-10_ELP_ coated on titanium discs, a cell adhesion assay was performed using crystal violet. The hMSCs were incubated for up to 150 min (in 30-min increments) in serum-free medium and adhered to the titanium discs with or without 10 μg·mL^−1^ of FN9-10_ELP_. As indicated in Fig 4, the adhesion rate of hMSCs in both groups was time-dependent and was 1.95-fold higher on the FN9-10_ELP_-titanium than on the control titanium (p < 0.001). As shown by the results, the adhesion rate was seen to increase during the initial 120 min and then became saturated. These results presented that the coating of titanium discs with FN9-10_ELP_ promoted the adhesion of hMSCs and that the optimal adhesion time is 120 min.

### Enhanced proliferation of hMSCs on the FN9-10_ELP_-coated titanium discs

**Fig 5:**
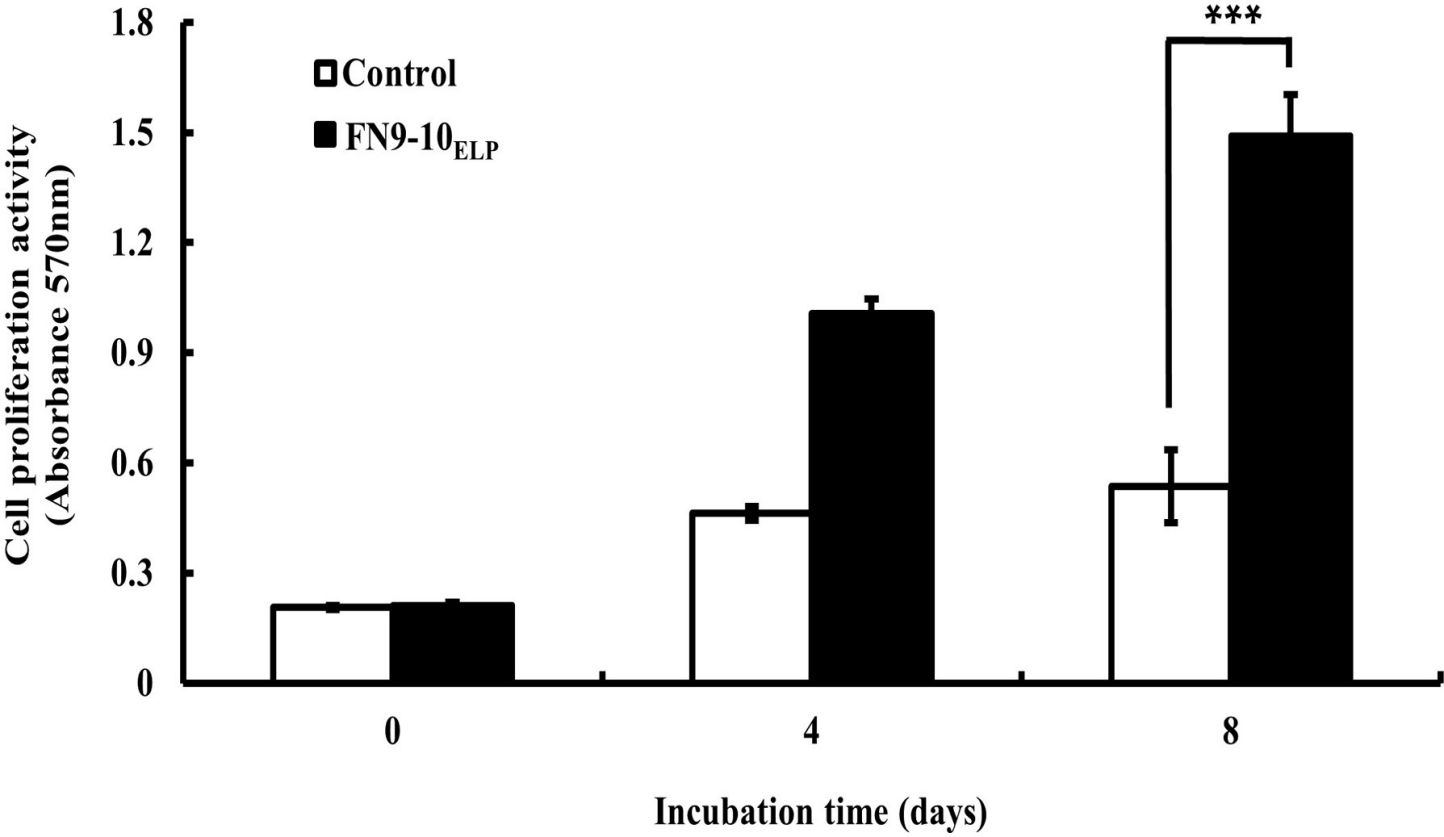
Cell proliferation activity of hMSCs on the FN9-10_ELP_-titanium discs for 0, 4 and 8 days. The titanium discs were immersed in 0 or 10 μg·mL^−1^ FN9-10_ELP_ overnight at 4°C then, hMSCs were seeded at a density of 1 × 10^4^ cells/disc on titanium discs and incubated for 0, 4 and 8 days at 37°C. The absorbance of formazan contained in the cells was used as a measure of cell proliferation. The cell proliferation activities are expressed as the mean ± SD (n = 3). p < 0.001.

To evaluate the hMSCs proliferative activity of FN9-10_ELP_ coated on titanium discs, cell proliferation was examined using a MTT assay. The results presented in Fig 5 indicated that the cell proliferation rate on the control discs was insignificant after 4 and 8 days of culture. However, the proliferation rate on the FN9-10_ELP_-titanium was significantly increased for up to 8 days (p < 0.001). Moreover, the proliferation of the hMSCs after 8 days on FN9-10_ELP_ titanium was 2.8-fold that on the control discs. These results demonstrated that the proliferation of hMSCs on titanium discs was enhanced by bio-functionalization with FN9-10_ELP_.

### Osteogenic differentiation activity of hMSCs on the FN9-10_ELP_-coated titanium discs

To investigate the effect of FN9-10_ELP_ for the osteogenic differentiation in protein level of hMSCs on titanium discs, alkaline phosphatase (ALP) assay and Alizarin Red S staining were performed.

**Fig 6:**
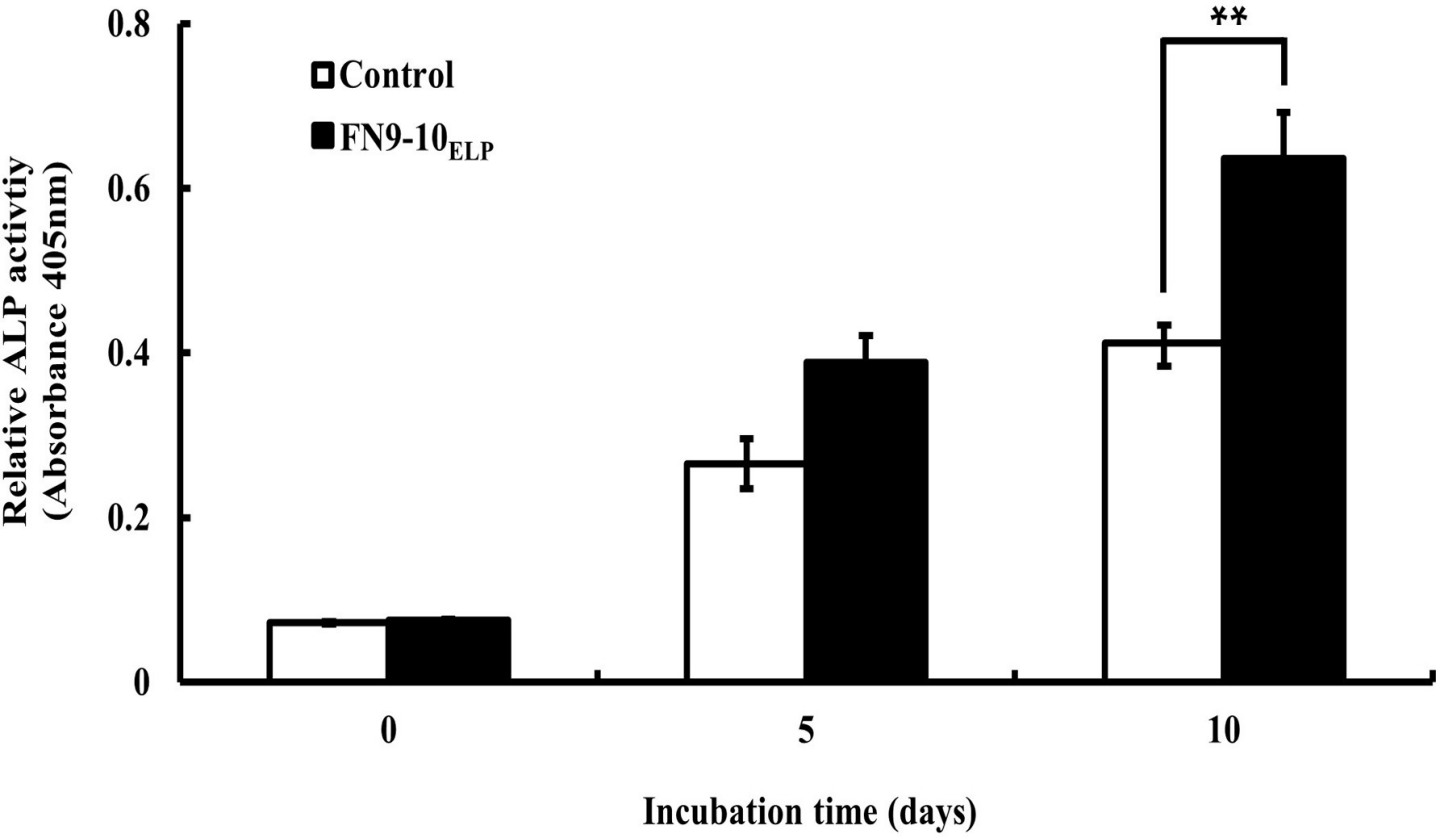
ALP activity of hMSCs on the FN9-10_ELP_-titanium discs for 0, 5 and 10 days. The titanium discs were immersed in 10 μg·mL^−1^ FN9-10_ELP_ overnight at 4°C, while the non-coated discs served as the control. The hMSCs were seeded at a density of 5 × 10^3^ cells/disc and incubated for 0, 5 and 10 days at 37°C. The ALP activities were normalized to the control and are reported as the mean ± SD (n = 3). p < 0.01.

ALP activity, an important early phase activity of osteogenic differentiation, was evaluated after incubating hMSCs on 0 or 10 μg·mL^−1^ FN9-10_ELP_-titanium for 10 days. As indicated in Fig 6, the ALP activity after 10 days was 1.55-fold higher for the hMSCs (cultured on FN9-10_ELP_-titanium) than on control titanium (p < 0.01).

**Fig 7:**
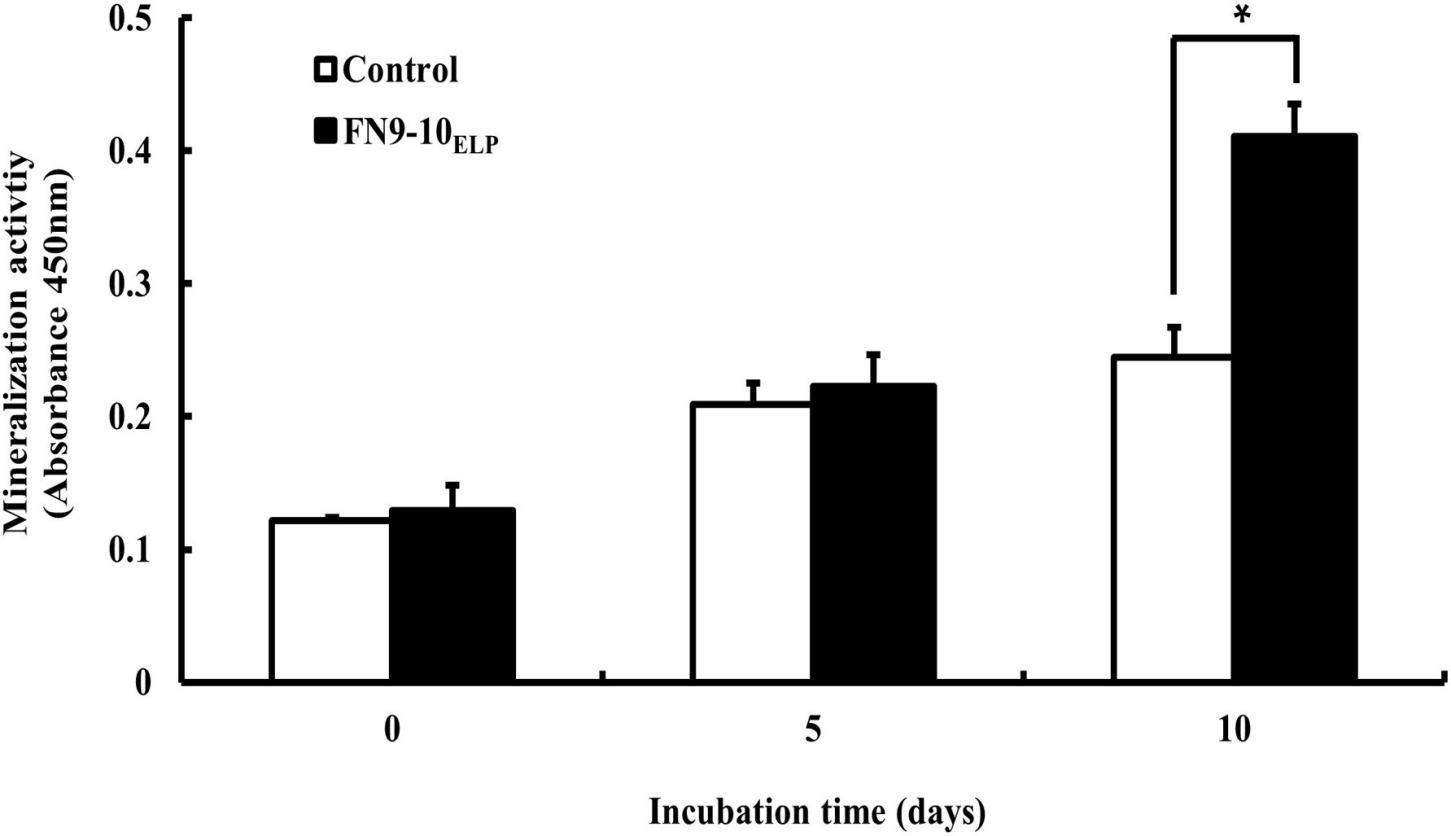
Mineralization activity of hMSCs on the FN9-10_ELP_-titanium discs for 0, 5 and 10 days. The titanium discs were immersed in 10 μg·mL^−1^ FN9-10_ELP_ overnight at 4°C, while the non-coated discs served as the control. The hMSCs were seeded at a density of 5 × 10^3^ cells/disc and incubated for 0, 5 and 10 days at 37°C. The mineralization activities are reported as the mean ± SD (n = 3). p < 0.05.

Mineralization activity, an important late phase activity of osteogenic differentiation, was examined after incubating hMSCs on 0 or 10 μg·mL^−1^ FN9-10_ELP_-titanium for 10 days. As shown in Fig 7, the mineralization activity after 10 days was 1.68-fold higher for the hMSCs (cultured on FN9-10_ELP_-titanium) than on non-coated titanium (p < 0.05). These results indicated that FN9-10_ELP_ treatment resulted in enhanced osteogenic differentiation of hMSCs on titanium discs.

### Evaluation of the expression for osteogenesis-related genes

**Fig 8:**
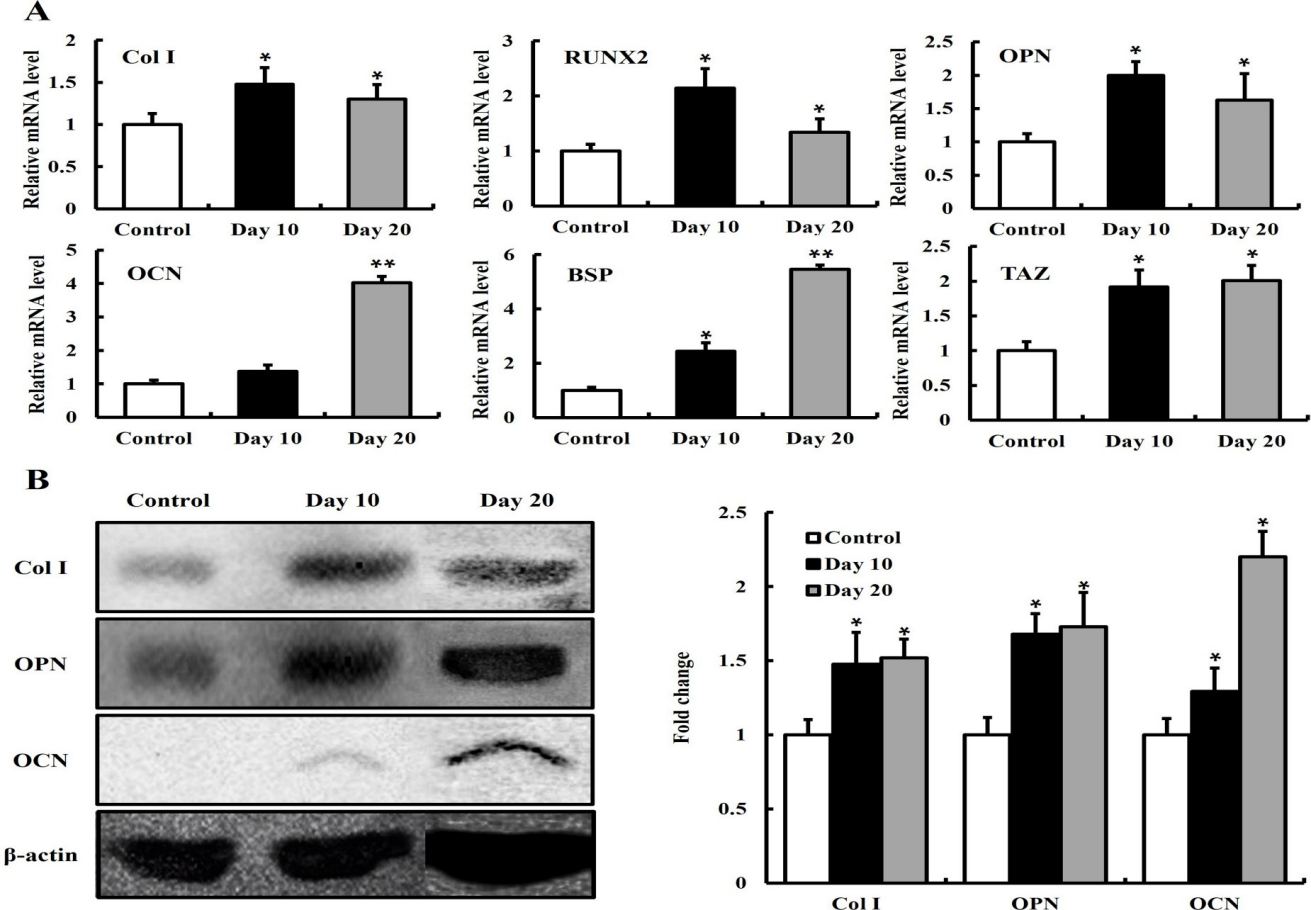
Osteogenic differentiation activities of hMSCs on the FN9-10_ELP_-titanium discs for 20 days. The titanium discs were immersed overnight in 10 μg·mL^−1^ of FN9-10_ELP_ at 4°C, while the non-coated discs served as the control. The hMSCs were seed at a density of 5 × 10^3^ cells/disc and incubated for 20 days at 37°C. (A) The mRNA levels of osteogenesis-related genes (Col I, RUNX2, OPN, OCN, BSP, TAZ) were analyzed via real-time PCR, and the comparative mRNA levels of each gene were evaluated relative to that of the β-actin as an internal control. The osteogenic differentiation activities are presented as the mean ± SD (n = 3). p < 0.05 and p < 0.01. (B) The protein levels of Col I, OPN, and OCN were analyzed via western blotting. Band density values of Col I, OPN, and OCN were normalized with β-actin and these proteins were represented as an arbitrary ratio compared to control titanium.

To further examine the osteogenic differentiation activity of hMSCs on the titanium discs with or without 10 μg·mL^−1^ FN9-10_ELP_, the mRNA expression levels of osteogenesis-related genes such as Col I, RUNX2, OPN, OCN, BSP, and TAZ were measured using the real-time- polymerase chain reaction (PCR) with β-actin as an internal control. As shown in Fig 8A, the mRNA levels of all five osteogenic markers extracted from the hMSCs were significantly up-regulated on the FN9-10_ELP_-titanium compared to the control. Treatment with FN9-10_ELP_ for 20 days upregulated significantly (p < 0.05) the mRNA expression of Col I (1.5-fold), RUNX2 (2.15-fold), OPN (2-fold), OCN (4.1-fold), and BSP (5.5-fold). Cui CB et al. reported that TAZ positively regulates bone formation *in vivo*, which seems to be mediated by the enhancement of both RUNX2 [42] and TGF-β [43] signaling. In this study, the treatment of hMSCs with FN9-10_ELP_ for 20 days resulted in significant (p < 0.05) upregulation of the TAZ (2-fold) genes. Thus, these results suggested that FN9-10_ELP_ treatment resulted in enhanced mRNA level of five osteogenic markers in hMSCs and TAZ might be involved in the activation of hMSCs associated with osteogenic differentiation.

In addition, the protein expressions of Col I, OPN, and OCN were analyzed by western blotting. As shown in Fig 8B, western blotting experiments showed the increase in the protein expression of Col I, OPN, and OCN extracted from the hMSCs induced with FN9-10_ELP_ for 20 days compared to the control (p < 0.05). Taken together, these results indicated that the expression of osteogenesis-related genes was upregulated in the presence of FN9-10_ELP_ compared with control titanium.

## Discussion

ECMs such as FN and ELP have been demonstrated to regulate osteogenesis-related cellular processes by signaling with MSC integrin [44,45]. In particular, FN is known to promote cell adhesion and proliferation activity of cell types such as MC3T3-E1 cells [46] and rBMSCs [12]. Recently, the biological properties of various stem cell types on ECM-modified titanium discs have been studied in the field of tissue engineering [47–49]. Lewallen EA et al. reported that modified titanium alters the biological phenotype of MSCs and modulates differentiation and ECM production [50]. However, most studies did not report the sustainability of bio-functionalization. In this study, we investigated sustainability of bioactivation as well as promoting hMSCs function by functionalizing titanium discs with a fusion FN9-10_ELP_ protein for more direct bone healing.

The first objective of this study was to evaluate the bio-functionalization and sustainability of the titanium surface. Therefore, we used an ELISA-based assay, a familiar method to evaluate the attachment and release of biomolecules from biomaterials [51]. In this study, the modification of the titanium surface with the FN9-10_ELP_ could be achieved at various concentration of FN9-10_ELP_ up to a maximum 10 μg (Fig 2). In addition, the release profile of FN9-10_ELP_ from the titanium discs revealed a sustained-release activity for up to 4 weeks without the initial burst effect that was observed for the control (Fig 3). Also, the amount of FN9-10_ELP_ retained on the titanium discs was about 42% for the approximately 10 days.

The next objective was to enhance the cellular functions of the hMSCs on the FN9-10_ELP_-titanium. From our study, it is evident that the adhesion activity of hMSCs increased with increasing incubation time on the FN9-10_ELP_-titanium up to maximum of 120 min and remained constant thereafter (Fig 4). Furthermore, the proliferation activity of the hMSCs on the FN9-10_ELP_-titanium was significantly stimulated compared to that on the control, especially at 8 days (Fig 5). In osteogenesis respect, ALP activity, an early-stage osteogenic differentiation, and mineralization, a late-stage osteogenic differentiation, were evaluated to investigate the osteoinduction effect of FN9-10_ELP_ [52,53]. We observed that FN9-10_ELP_-titanium upregulated the osteogenic differentiation activities of hMSCs in protein level compared to control at 10 days (Figs 6 and 7). These results are in close agreement with a few previous studies, wherein, an increase in adhesion, proliferation, and osteogenic differentiation activities is observed due to supporting biological environment of fusion ECM protein [22,40].

To further characterize the osteogenic differentiation of the hMSCs on the FN9-10_ELP_-titanium, the mRNA levels of osteogenesis-related markers such as Col I, RUNX2, OPN, OCN, BSP, and TAZ were examined via real-time PCR. In this study, mRNA expressions of Col I and RUNX2 involved in the early-stage of osteogenesis [53–56], are upregulated in hMSCs induced on the FN9-10_ELP_-titanium for 10 days compared to that on control and is gradually down-regulated (Fig 8A). The mRNA expression of OCN, BSP, and OPN, involved in the late-stage of osteogenesis [57–59], are upregulated in hMSCs induced on the FN9-10_ELP_-titanium for 20 days compared to that on control. The mRNA expression of TAZ is also upregulated for 20 days. In addition, results of western blotting indicated the increased protein expression of Col I, OPN, and OCN on FN9-10_ELP_-titanium treated hMSCs confirming its osteogenic potential (Fig 8B). Recently, Laino L et al. conducted to improve the osteogenesis of stem cells by modifying the titanium surface and showed that the Col I expression was increased in early-osteogenesis (matrix deposition) and the expression of BSP and OPN was increased in late-osteogenesis (matrix mineralization) [60]. Liu Jet al. investigated osteoblast differentiation by modifying antibacterial osteogenic peptides on titanium and showed that RUNX2 as one of the master transcription factors enhances the expression of downstream genes (e.g., Col I, OCN, etc.) [61]. Cui CB et al. reported that TAZ function as a transcriptional coactivator may promote be the expression of the bone-related genes such as OCN in a manner that seems to be regulated by RUNX2 signaling [42]. In addition, La Noce M et al. reported that YAP/TAZ pathway activation by hyaluronan in DPSCs can promote bone differentiation and improve mineralization by expression of bone-related markers [62]. Taken together, these results are consistent with our present study. Therefore, the authors found that the cellular function of hMSCs for bone healing was enhanced in *in vitro* modified titanium, suggesting that the microenvironment required for osteogenic differentiation was continuously provided.

## Conclusion

In conclusion, the present study has demonstrated that the bio-functionalization of titanium discs with FN9-10_ELP_ can effectively promote the cell adhesion, proliferation, and osteogenic differentiation of hMSCs and sustain bio-functionalization during osteogenic differentiation. These results showed that sustained biological activity of FN9-10_ELP_ released from titanium over the full 4-week period might improve bone regeneration, healing and suggest potential use for implant treatment.

## Supporting information

S1 File (XLSX)
